# Polymorphisms of ATG5 Gene Are Associated with Autoimmune Thyroid Diseases, Especially Thyroid Eye Disease

**DOI:** 10.1155/2022/3881417

**Published:** 2022-04-26

**Authors:** Wen Wang, Zheng-yao Yu, Rong-hua Song, Shuang-tao He, Liang-feng Shi, Jin-an Zhang

**Affiliations:** ^1^Department of Endocrinology, Shanghai University of Medicine & Health Sciences Affiliated Zhoupu Hospital, No. 1500 Zhouyuan Road, Pudong District, Shanghai, China 201318; ^2^Department of Endocrinology, Jinshan Hospital of Fudan University, No. 1508 Longhang Road, Jinshan District, Shanghai, China 201508; ^3^Department of Otolaryngology, Shanghai Songjiang District Central Hospital, No. 746 Middle Zhongshan Road, Shanghai, China 201600

## Abstract

**Objective:**

To explore the association of *ATG5* gene polymorphisms with autoimmune thyroid diseases (AITDs) including Hashimoto's thyroiditis (HT) and Graves' illness (GD) as well as their clinical features.

**Methods:**

rs6568431, rs548234, and rs6937876 were selected to investigate the correlation of single-nucleotide polymorphisms of *ATG5* gene with AITDs. Their frequencies in 824 AITD patients, including 271 HT patients and 553 GD patients, and 764 healthy controls were tested using both ligase detection reaction and multiplex polymerase chain reaction.

**Results:**

Allele A frequency of rs6568431 in AITDs patients (*p* = 0.016, OR = 1.201, 95% CI = 1.034 − 1.394) and allele G frequency of rs6937876 in AITDs patients (*p* = 0.009, OR = 1.223, 95% CI = 1.052 − 1.422) and in GD patients (*p* = 0.009, OR = 1.247, 95% CI = 1.056 − 1.473) were significantly higher than those in the healthy controls. The frequency of G allele (*p* = 5.42E − 18, OR = 0.242, 95% CI = 0.173 − 0.339) of rs6937876 was significantly higher in GD patients with ophthalmopathy. However, no relationship was found between family history, age onset, and the three SNPs.

**Conclusion:**

The study is the first to reveal the association between AITDs and *ATG5* polymorphisms, and *ATG5* gene is considered as a predisposing gene to AITDs, especially GDs.

## 1. Introduction

Autoimmune endocrine diseases are organ-specific diseases targeting endocrine glands including the islet cells, ovary, thyroid, and adrenal glands. Among them, autoimmune thyroid diseases (AITDs), which primarily include Hashimoto's thyroiditis (HT) and Graves' illness (GD) in humans, are one of the most widely spread autoimmune endocrine illnesses [1] and the major cause of thyroid gland dysfunctions [2]. Although environmental factors such as mental pressure, infection, medication, smoking habits, and iodine are reported to be involved in AITDs, genes with AITD susceptibility have been characterized and identified including *non-MHC genes* (such as *PTPN22*, *thyroglobulin*, *CD40*, *CTLA-4*, and *TSH receptor genes*) and *HLA-DR gene* [1]. With the progress of genome-wide association studies (GWAS) in recent years, an increasing number of genes have been found to be associated with AITDs.

Autophagy, an evolutionarily conservative procedure, is of great significance in helping recycle organelles and remove useless cytoplasmic cargo [3]. Recent studies have shown that autophagy not only participates in antigen processing and presentation but also promotes lymphocyte production of IL-6, IL-8, and other cytokines. The initiation and formation of autophagosome require two ubiquitin-like conjugation pathways. In both systems, autophagy-related gene (Atg) 5 plays a pivotal role [3]. *ATG5* was first identified in Burkitt's lymphoma apoptotic cells and is located in human chromosome 6q21. Human *ATG5* contains 275 amino acid residues with a molecular weight of approximately 32.4 kDa [4]. Previous studies have revealed that *ATG5* polymorphisms are linked to a wide range of immune-mediated and autoimmune diseases, including multiple sclerosis [5], systemic lupus erythematosus [6], and rheumatoid arthritis [7]. Since most autoimmune diseases have similar pathogenesis and are related to various common genetic variations, we proposed a hypothesis that some *ATG5* polymorphisms might be genetic factors correlated with potential AITDs. Therefore, we undertook this case-control association analysis involving 1588 participants (824 patients with AITDs and 764 controls) in an attempt to identify these *ATG5* polymorphisms through the approach of candidate gene association.

## 2. Participants and Methods

### 2.1. Participants

In this case-control study, 824 AITD Han Chinese patients were recruited from the Department of Endocrinology in Shanghai University of Medicine and Health Sciences Affiliated Zhoupu Hospital during 2016 and 2019. Among them, as Table 1 shows, 553 were GD patients aged 37.05 ± 14.57 years and 271 were HT patients aged 34.85 ± 13.86 years. GD patients were diagnosed by clinical manifestations, imaging examination, and laboratory tests of hyperthyroidism including circulating TSH receptor antibody (TRAb). HT patients were diagnosed based on the presence of an enlarged thyroid and antithyroglobulin (TgAb) or antithyroid peroxidase antibody (TPOAb) regardless of the presence of documented clinical or biochemical hypothyroidism with the help of the presence of diffusive thyroid changes in hypoechogenic areas in ultrasonic examination. Among the 553 GD patients, 93 had ophthalmopathy, 114 had family history, 66 had smoking history, and 98 had normal thyroid size. Among the 271 HT patients, 2 had ophthalmopathy, 58 had family history, 35 had smoking history, and 37 had normal thyroid size. In this study, ophthalmopathy is regarded as a distinctive disease characterized clinically or radiographically by inflammation and swelling of extraocular muscles, orbital fat, periorbital edema, exophthalmos, scleral injection, eyelid constriction, and swelling of conjunctions [2]. On examination, patients may have staring gaze, lid lag, signs of conjunctiva inflammation, periorbital edema, and abnormalities of conjugate gaze. Orbital imaging with CT scanning may find extraocular muscle enlargement. AITD family history was defined as presence of AITDs in the participants' first-degree relatives such as children, siblings, and parents and second-degree relatives including uncles, aunts, and grandparents.

In addition, 764 ethnically matched healthy participants were recruited from the Health Check-Up Center of Shanghai University of Medicine and Health Sciences Affiliated Zhoupu Hospital during the same period and screened for the absence of palpable goiter and negative TPOAb.

This study was approved by the Ethics Committee of Shanghai University of Medicine and Health Sciences Affiliated Zhoupu Hospital (2018-C-027-E0). Written informed consents were provided by the participants or their parents.

### 2.2. DNA Extraction and SNP Genotyping

Genomic DNA was extracted from 1 mL of peripheral blood of each participant using the RelaxGene Blood DNA System (Tiangen Biotech, Co., Beijing, China) in accordance with the manufacturer's protocol. Three SNPs of *ATG5*, rs6568431, rs548234, and rs6937876, were selected from HapMap CHB database using Haploview software 4.2 according to the following criteria: Hardy-Weinberg equilibrium (HWE) > 0.05, minor allele frequency (MAF) > 0.05, and logarithm of odds (LOD) > 3.0. The three SNPs are located in the intergenic region of the ATG5-PRDM1, which is an autoimmune disease-associated locus that plays an important role in immune regulation [8]. Genotyping of rs6568431, rs548234, and rs6937876 was performed using ligase detection reactions (LDR) platform (Illumina NovaSeq 6000, USA), and the targeted DNA sequences were amplified using multiplex polymerase chain reaction (PCR) with specific primer sets shown in Table 2.

### 2.3. Statistical Analysis

Clinical data were presented as mean ± standard deviation (SD) and analyzed by using the SPSS 21.0 software (IBM, Chicago, USA). Differences in SNP frequencies between the disease and the control groups were analyzed using chi-squared test. Association of different genotypes with ophthalmopathy in GD group, AITD family history, and age was analyzed using candidate gene association analysis and Pearson's chi-squared test. Odds ratio (OR) was to assess the correlation between each SNPs and AITD. A Bonferroni adjustment for multiple comparisons was used, with *p* < 0.017 for statistical significance.

## 3. Results

### 3.1. Allele and Genotype Results

In both case group and control group, all SNPs were in HWE (*p* > 0.05). The case-control association analysis and allele and genotype frequencies for each SNP are shown in Table 3. The results showed that allele A of rs6568431 was present more in AITD patients than in the controls (*p* = 0.016, OR = 1.201, 95% CI = 1.034 − 1.394), while its frequency was not significantly different between HT patients and controls (*p* = 0.052, OR = 1.229, 95% CI = 0.998 − 1.513). Similarly, allele G of rs6937876 was present more in AITD and GD patients than in the controls (*p* = 0.009, OR = 1.223, 95% CI = 1.052 − 1.422 and *p* = 0.009, OR = 1.247, 95% CI = 1.056 − 1.473, respectively). Nevertheless, no significant differences between AITD patients and controls were found in the frequency of both alleles of rs548234. Furthermore, when analyzed separately, no notable differences were found in genotype frequency of rs6937876, rs548234, and rs6568431 between the control group and HT patients or GD patients, as shown in Table 4.

### 3.2. Genotype and Clinical Phenotype Correlations

Table 5 shows the allele frequencies of rs6568431, rs548234, and rs6937876 in ophthalmopathy and nonophthalmopathy GD patients. Compared with that of healthy participants, allele G of rs6937876 was present more in GD patients with ophthalmopathy (*p* = 5.42E − 18, OR = 0.242, 95% CI = 0.173 − 0.339).

No significant difference was identified between family history, age onset, and the three SNPs (*p* > 0.017; data not shown).

## 4. Discussion

Autophagy is a lysosome-mediated metabolic procedure to maintain intracellular homeostasis. It is achieved through the circulation and degradation of organelles as well as cytoplasmic components [9]. Generally, autophagy can protect cellular functions and allow cells to adapt to certain stress conditions including chronic stimulation, intracellular accumulation of damaged proteins, oxidative stress, and nutrient starvation. Autophagy plays an important role in sensing cytoplasmic danger signals including nucleic acids, which are common inducers of inflammatory responses and targets of autoimmune responses [10].

Physically, autophagy is a process for cells to destroy detrimental proteins and organelles by lysosomes. This entire process relies on a series of autophagy-related gene (atg) products to complete. A large amount of existing genetic researches have revealed that rare variants of ATG5 are associated with cancers including colon cancer, stomach cancer, and prostate cancer, Parkinson disease, and other complex illnesses [11–13]. Studies have also shown that ATG5 gene may act as a potential risk factor to promote the occurrence of autoimmune diseases. SNPs of rs6568431, rs548234, and rs6937876 loci have been proven to be associated with various autoimmune diseases. For example, rs548234 has been associated with rheumatoid arthritis and multiple sclerosis in Caucasian population [7]. Investigators have reported upregulation of ATG5 protein in T cell-infiltrated inflammatory areas of patients with rheumatoid arthritis [5], and ATG5 variants are associated with neuromyelitis optica in southern Chinese Han population [8]. Other researches have also found some correlation of ATG5 gene variants with SLE in Caucasian population [14, 15] and significant positive correlations of ATG5 expression with systemic lupus erythematosus in Chinese Han population. Moreover, in vitro experiments have also confirmed the regulatory role of rs6937876 in B cell populations [6]. But to date, the association between ATG5 SNPs and AITDs has not been reported.

The case-control study attempted to determine the association between polymorphisms of *ATG5* and AITDs. The results showed that allele A frequency of rs6568431 and allele G frequency of rs6937876 were significantly higher in patients with AITDs, which is similar to a finding that their frequency is also higher in patients with SLE [16], a classic autoimmune disease, suggesting that *ATG5* SNPs could lead to higher susceptibility to autoimmune diseases. In addition, the study also found that GD patients with G allele of rs6937876 had an increased susceptibility of ophthalmopathy development.

Although the etiology of AITDs is incompletely understood, dysregulated immune response system is known to be involved in various perspectives. For example, dysregulation of effector T cell responses participates in the occurrence and development of AITDs. Autophagy is involved in both extracellular and intracellular antigen processing for MHC class II presentation to CD4^+^ T cells [17]. Regardless of negative or positive selections, thymic epithelial cells play an important role in endogenous antigen presentation in an MHC II-restricted fashion, as explained in the proposed model of how autophagy affects T cell repertoires [18]. All specificities of CD8^+^ T cells and some particular positive specificities of CD4^+^ T cells were observed in athymic recipients or TCR transgenic laboratory mice after *ATG5*^−/−^ thymi transplantation, suggesting that T cell repertoire was shaped towards multiorgan inflammation due to the deficiency of *ATG5* [19]. Moreover, laboratory mice with dendritic cell-conditional deletion in *ATG5* displayed obvious deficiency or inability to process and present phagocytosed antigens including toll-like receptor stimuli for MHC class II [20]. Therefore, *ATG5* may impact the processing of toll-like receptor stimuli for MHC class II and affect the procedure of presenting MHC class II to CD4^+^ T cells, which consequently will suppress the T cell signaling transduction and activation.

We also investigated the association between genotype and clinical phenotype. We noticed that allele G of rs6937876 was more common in GD patients with ophthalmopathy. Since ATG5 is a candidate gene for the ATG5-PRDM1 locus, the possible functional variant rs6937876 may contribute to the pathogenesis of GO in multiple ways. Autophagy is necessary for maintaining ocular immune privilege, and the deletion of multiple autophagy genes in macrophages leads to inflammation-mediated eye disease [21]. Overall, *ATG5* gene mutations may be connected with illness predisposition by influencing targeted gene expression or protein structures.

The study has some limitations. First, we only investigated three SNPs of *ATG5* gene. The roles of these three SNPs in AITDs and other *ATG5* SNPs as well as their association with AITDs need to be further investigated in a wider range of ethnicities. Second, we calculated the power. The power of the data was evaluated by the Power and Sample Size Calculation Software [22]. The allele frequency of rs6937876 was the lowest among the three SNPs, which is 19% according to the HapMap data. Considering the expected frequency of minor allele of rs6937876 in the controls, the combined set of 553 GD patients and 764 controls gave a power of 34.9% with an OR = 1.24 at the 5% significance level. Therefore, we need to collect more samples to ensure effective power in further research.

## 5. Conclusion

We reported for the first time the contribution of *ATG5* variants to GD susceptibility. The variant rs6937876 reside in PRDM1 and ATG5 region and is strongly associated with the susceptibility to thyroid-related eye diseases. Taking the contradictory outcomes of *ATG5* gene polymorphisms in various ethnicities into consideration, more research is needed to explore the function of *ATG5* in the pathogenesis of AITDs.

## Figures and Tables

**Table 1:** Clinical data of AITD patients and controls.

	GD	HT	Control
Number	553	271	764
Gender			
Male	171	37	268
Female	382	234	496
Age	37.05 ± 14.57	34.85 ± 13.86	39.20 ± 8.74
Family history			
(+)	114	58	
(-)	395	201	
Thyroid size			
Normal	98	37	
First degree	94	45	764
Second degree	294	170	
Third degree	67	19	
Ophthalmopathy			
(+)	93	2	
(-)	460	269	
Smoking			
(+)	66	35	342
(-)	487	236	422

**Table 2:** Primers used for multiplex PCR.

SNP	Primer	Sequences (5′-3′)
rs6568431	Forward	TGAGCAAGGGCACTTCTAGG
	Reverse	TCTTGAACTCCGGACCTTGT
rs548234	Forward	GGAATAGACTACAAATCACACTCCA
	Reverse	TCAATCTCTTGCGCTCTTCA
rs6937876	Forward	CCCAAGGAACTGTCATGGAG
	Reverse	TGCCTTGAAGTCCTGAAAGAG

**Table 3:** Allele and genotype frequencies in patients and controls.

SNP ID		Allele/genotype frequency *n* (%)				*P* value (OR)		
		Control (%)	AITD (%)	GD (%)	HT (%)	AITDs vs. C	GD vs. C	HT vs. C
rs6568431	A	461 (30.17)	563 (34.16)	375 (33.91)	188 (34.69)	0.016 (1.201)	0.042(1.187)	0.052 (1.229)
	C	1067 (69.83)	1085 (65.84)	731 (66.09)	354 (65.31)			
	AA	67 (8.76)	88 (10.68)	59 (10.57)	29 (10.70)	0.044	0.114	0.124
	AC	327 (42.80)	387 (46.97)	257 (46.47)	130 (47.97)			
	CC	370 (48.44)	349 (42.35)	237 (42.76)	112 (41.33)			

rs548234	C	382 (25.00)	455 (27.61)	307 (27.76)	148 (27.31)	0.095 (1.144)	0.112 (1.153)	0.052 (1.229)
	T	1146 (75.00)	1193 (72.39)	799 (72.24)	394 (72.69)			
	CC	53 (6.94)	64 (7.77)	43 (7.78)	21 (7.75)	0.213	0.242	0.124
	CT	276 (36.13)	327 (39.68)	221 (39.96)	106 (39.11)			
	TT	435 (56.93)	433 (52.55)	289 (52.26)	144 (53.14)			

rs6937876	G	444 (29.05)	550 (33.37)	374 (33.82)	176 (32.47)	0.009 (1.223)	0.009 (1.247)	0.136 (1.174)
	A	1084 (70.95)	1098 (66.63)	732 (66.18)	366 (67.53)			
	GG	70 (9.16)	89 (10.80)	61 (11.03)	28 (10.33)	0.020	0.023	0.277
	AG	304 (39.80)	372 (45.15)	252 (45.57)	120 (44.28)			
	AA	390 (51.04)	363 (44.05)	240 (43.40)	123 (45.39)			

**Table 4:** Comparisons of genotype frequencies in AITD patients and controls.

SNP ID		Allele/genotype frequency *n* (%)				*P* value [OR (95% CI)]		
		Control	AITD	GD	HT	AITDs vs. C	GD vs. C	HT vs. C
rs6568431	AA	67 (8.7)	88 (10.7)	59 (10.7)	29 (10.7)	0.200 [1.244 (0.890-1.737)]	0.247 [1.242 (0.860-1.796)]	0.346 [1.247 (0.787-1.974)]
	AC+CC	697 (91.3)	736 (89.3)	494 (89.3)	242 (89.3)	Reference	Reference	Reference

rs548234	CC	53 (6.9)	65 (7.9)	43 (7.8)	21 (7.7)	0.474 [1.147 (0.787-1.673)]	0.563[1.131 (0.745-1.718)]	0.656 [1.127 (0.666-1.906)]
	CT+TT	711 (93.1)	760 (92.1)	510 (92.2)	250 (92.3)	Reference	Reference	Reference

rs6937876	GG	70 (9.2)	89 (10.8)	61 (11.0)	28 (10.3)	0.277 [1.201 (0.863-1.670)]	0.263 [1.229 (0.856-1.766)]	0.572 [1.142 (0.720-1.813)]
	AG+AA	694 (90.8)	735 (89.2)	492 (89.0)	243 (89.7)	Reference	Reference	Reference

**Table 5:** Allele frequencies of rs6568431, rs548234, and rs6937876 in ophthalmopathy and nonophthalmopathy GD patients.

SNP	Alleles	Ophthalmopathy (%)	Nonophthalmopathy (%)	*P* value	OR	95% CI
rs6568431	A	61 (32.80)	314 (34.13)	0.762	0.942	(0.674-1.317)
	C	125 (67.20)	606 (65.87)			
	AA	8 (8.60)	51 (11.09)	0.479	0.755	(0.346-1.648)
	AC+CC	85 (91.40)	409 (88.91)			

rs548234	C	48 (25.81)	259 (28.15)	0.515	0.888	(0.620-1.270)
	T	138 (74.19)	661 (71.85)			
	CC	3 (3.23)	40 (8.69)	0.072	0.350	(0.106-1.156)
	CT+TT	90 (96.77)	420 (91.31)			

rs6937876	G	59 (31.72)	315 (34.24)	5.42E-18	0.242	(0.173-0.339)
	A	127 (68.28)	605 (65.76)			
	GG	7 (7.53)	54 (10.66)	0.237	0.612	(0.269-1.391)
	AG+AA	86 (92.47)	406 (88.26)			

## Data Availability

All data generated or analyzed during this study are included in this article.

## References

[1] Huber A., Menconi F., Corathers S., Jacobson E. M., Tomer Y. (2008). Joint genetic susceptibility to type 1 diabetes and autoimmune thyroiditis: from epidemiology to mechanisms. *Endocrine Reviews*.

[2] Vanderpump M. P., Tunbridge W. M., French J. M. (1995). The incidence of thyroid disorders in the community: a twenty-year follow-up of the Whickham survey. *Clinical Endocrinology*.

[3] Huett A., Xavier R. J. (2010). Autophagy at the gut interface: mucosal responses to stress and the consequences for inflammatory bowel diseases. *Inflammatory Bowel Diseases*.

[4] Ye X., Zhou X. J., Zhang H. (2018). Exploring the role of autophagy-related gene 5 (ATG5) yields important insights into autophagy in autoimmune/autoinflammatory diseases. *Frontiers in Immunology*.

[5] Alirezaei M., Fox H. S., Flynn C. T. (2009). Elevated ATG5 expression in autoimmune demyelination and multiple sclerosis. *Autophagy*.

[6] Zhou X. J., Lu X. L., Lv J. C. (2011). Genetic association of PRDM1-ATG5 intergenic region and autophagy with systemic lupus erythematosus in a Chinese population. *Annals of the Rheumatic Diseases*.

[7] Raychaudhuri S., Thomson B. P., Remmers E. F. (2009). Genetic variants at *CD28*, *PRDM1* and *CD2/CD58* are associated with rheumatoid arthritis risk. *Nature Genetics*.

[8] Lu X. L., Zhou X. J., Guo J. P. (2011). Rs548234 polymorphism at PRDM1-ATG5 region susceptible to rheumatoid arthritis in Caucasians is not associated with rheumatoid arthritis in Chinese Han population. *Chinese Medical Journal*.

[9] Ravikumar B., Sarkar S., Davies J. E. (2010). Regulation of mammalian autophagy in physiology and pathophysiology. *Physiological Reviews*.

[10] Yang Z., Goronzy J. J., Weyand C. M. (2015). Autophagy in autoimmune disease. *Journal of Molecular Medicine (Berlin, Germany)*.

[11] Kim M. S., Song S. Y., Lee J. Y., Yoo N. J., Lee S. H. (2011). Expressional and mutational analyses of ATG5 gene in prostate cancers. *APMIS*.

[12] An C. H., Kim M. S., Yoo N. J., Park S. W., Lee S. H. (2011). Mutational and expressional analyses of ATG5, an autophagy-related gene, in gastrointestinal cancers. *Pathology, Research and Practice*.

[13] Kang M. R., Kim M. S., Oh J. E. (2009). Frameshift mutations of autophagy-related genes ATG2B, ATG5, ATG9B and ATG12 in gastric and colorectal cancers with microsatellite instability. *The Journal of Pathology*.

[14] Harley J. B., Alarcón-Riquelme M. E., Criswell L. A. (2008). Genome-wide association scan in women with systemic lupus erythematosus identifies susceptibility variants in *ITGAM*, *PXK*, *KIAA1542* and other loci. *Nature Genetics*.

[15] Gateva V., Sandling J. K., Hom G. (2009). A large-scale replication study identifies TNIP1, PRDM1, JAZF1, UHRF1BP1 and IL10 as risk loci for systemic lupus erythematosus. *Nature Genetics*.

[16] Hui-Yuen J. S., Zhu L., Wong L. P. (2016). Chromatin landscapes and genetic risk in systemic lupus. *Arthritis Research & Therapy*.

[17] Dengjel J., Schoor O., Fischer R. (2005). Autophagy promotes MHC class II presentation of peptides from intracellular source proteins. *Proceedings of the National Academy of Sciences of the United States of America*.

[18] Nedjic J., Aichinger M., Emmerich J., Mizushima N., Klein L. (2008). Autophagy in thymic epithelium shapes the T-cell repertoire and is essential for tolerance. *Nature*.

[19] Zhou X. J., Zhang H. (2012). Autophagy in immunity: implications in etiology of autoimmune/autoinflammatory diseases. *Autophagy*.

[20] Lee H. K., Mattei L. M., Steinberg B. E. (2010). In vivo requirement for Atg 5 in antigen presentation by dendritic cells. *Immunity*.

[21] Santeford A., Wiley L. A., Park S. (2016). Impaired autophagy in macrophages promotes inflammatory eye disease. *Autophagy*.

[22] Dupont W. D., Plummer W. D. (1998). Power and sample size calculations for studies involving linear regression. *Controlled Clinical Trials*.

